# Évolution des violences basées sur le genre lors de l’émergence de la pandémie de COVID-19 dans le Grand Abidjan (Mars 2020-Mai 2021)

**DOI:** 10.48327/mtsi.V2I2.2022.240

**Published:** 2022-06-08

**Authors:** Solange AMÉTHIER, Savané Sita KROMAN, Baba SANGARÉ, Djibril CHÉRIF, Florence KADJO KOUADIO, Daouda COULIBALY, Bi Vroh Joseph BÉNIÉ

**Affiliations:** 1 Programme national de prise en charge des orphelins et autres enfants rendus vulnérables du fait du VIH (PN-OEV), BP 1816 Abidjan 08, Côte d'Ivoire; 2 Institut national d'hygiène publique (INHP), BPV 14 Abidjan, Côte d'Ivoire

**Keywords:** Violences basées sur le genre, Covid-19, Grand Abidjan, Afrique subsaharienne, Gender-based violence, Covid-19, Greater Abidjan area, Sub-Saharan Africa

## Abstract

Une enquête du ministère de la Famille a montré en 2018 l'importance des violences basées sur le genre (VBG) dans l'agglomération du Grand Abidjan (5 millions d'habitants). Les travailleurs sociaux employés pour ce travail ont été mobilisés lors de la pandémie de Covid-19 pour sensibiliser la population et l'aider dans la lutte contre le SARS-CoV-2 tout en poursuivant leur action contre les VBG. Ce sont les résultats recueillis qui font l'objet de cet article. Le nombre de viols est passé de 41 pour la période de janvier 2019 à février 2020 à 77 pour la période de mars 2020 à avril 2021, celui des agressions sexuelles de 4 à 7 et celui des agressions physiques de 139 à 171. Cette évolution n'est pas liée à celle de la Covid-19 ni à la commune concernée.

## Abstract (en)

In Côte d'Ivoire, a country of 28 million inhabitants in West Africa, a survey conducted by the Ministry of the Family in 2018 showed the extent of gender-based violence (GBV) in the Greater Abidjan area (5 million inhabitants), the country's economic capital. The social workers employed for this work were mobilized during the Covid-19 pandemic to raise awareness and help the population fight against SARS-CoV-2. The results collected by these workers during the first period (January 2019 - February 2020) and the second period (March 2020 - April 2021) are the subject of this article.

From the first period to the second, the number of rapes increased from 41 to 77, sexual assaults from 4 to 7, physical assaults from 139 to 171, and forced marriages from 4 to 7. In total, the social workers helped 303 victims during the pre-pandemic period and 402 during the pandemic. This evolution varies from municipality to municipality in the Greater Abidjan area, and is not related to the number of inhabitants per municipality and without any link with the frequency of Covid-19 in each municipality. Despite possible biases, this study highlights a major health problem, GBV, and demonstrates the probable negative effects of the pandemic.

## Introduction

La Côte d'Ivoire, pays d'Afrique de l'Ouest, compte 28 millions d'habitants en 2021, dont 5 millions dans le district de la capitale économique Abidjan [4]. Le premier cas de Covid-19 a été déclaré dans le pays le 11 mars 2020. Le 30 septembre 2020 la Côte d'Ivoire avait déclaré 19 724 cas et 120 décès et le 17 avril 2021, 45 560 cas et 274 décès, soit une létalité de 0,6 % [2].

Une enquête nationale sur les violences faites aux jeunes en Côte d'Ivoire, réalisée entre avril et septembre 2018, avait montré l'importance des violences faites aux enfants et aux jeunes. Ainsi, chez les 18-24 ans, 19,2 % des filles et 11,4 % des garçons ont subi une violence sexuelle; 47,1 % des filles et 60,8 % des garçons ont subi une violence physique. En outre, 19,0 % des filles et 15,5 % des garçons ont subi une violence émotionnelle avant leurs 18 ans [3].

Dès 2019, des travailleurs sociaux du ministère de la Femme, de la Famille et de l'Enfant ont alors été mobilisés dans les districts sanitaires du Grand Abidjan pour prendre en charge tous les patients qui signalaient une violence basée sur le genre (VBG). À cette occasion, ils remplissaient un questionnaire détaillé sur les faits et suivaient ensuite ces victimes sur les plans médical, psychosocial, matériel et juridique.

Dans le cadre de la riposte à la pandémie de Covid-19, le Centre des opérations d'urgence de santé publique (COUSP), organe interministériel dont l'objectif est d'alerter, analyser et proposer des actions pour tout problème de santé, a fait intervenir ces travailleurs sociaux dans les centres de santé et les centres de dépistage de Covid-19 pour expliquer et faciliter l'application des mesures de prévention contre le SARS-CoV-2.

L'objectif de cette étude était de recenser les informations recueillies par ces travailleurs sociaux surles VBG et d’évaluer les évolutions dans le temps entre 2 périodes successives, pré-pandémique puis pandémique, et d'en comparer les résultats afin d'objectiver une éventuelle influence de la crise sanitaire liée à la Covid-19 sur l'incidence des VBG.

## Méthodes

Quarante-six (46) travailleurs sociaux (ce terme général regroupe dans cette étude les éducateurs spécialisés, les assistants sociaux et les éducateurs préscolaires) issus de 8 complexes socio-éducatifs du Grand Abidjan ont été mobilisés pour mener cette activité dans les districts sanitaires correspondant au Grand Abidjan. Les éducateurs spécialisés mènent principalement des actions pour aider les personnes en difficulté à agir sur elles-mêmes ou leur environnement. Les assistants sociaux aident les personnes à s'adapter aux changements de la société. Les éducateurs préscolaires organisent des activités collectives et personnelles pour les jeunes enfants. Les complexes socio-éducatifs sont chargés de la mise en œuvre de la politique de protection de l'enfant, d'autonomisation des femmes et de promotion de la famille. Au nombre de 8, ils couvrent les communes, ou groupes de communes, de Koumassi, Port-Bouët, Treichville et Marcory, Adjamé et Attécoubé, Anyama, Abobo, Yopougon, Cocody et Bingerville. Les communes du Plateau et de Songon ne sont pas incluses dans cette étude.

Pendant la pandémie, outre des activités de communication et de sensibilisation, ces travailleurs sociaux ont accompagné les équipes médicales lors de l'annonce de cas confirmés de Covid-19 et de leur transfert sur les sites de prise en charge, et ont apporté assistance et accompagnement aux cas et à leurs contacts présentant un état de vulnérabilité.

Simultanément le recueil des cas de VBG, mis en place depuis 2019, a été réalisé avec un soutien social, psychologique, juridique et matériel. À cette fin, une fiche formulaire (guide d'entretien en annexe) a servi à recueillir les informations sur les VBG.

Les données ont été collectées pour la première période du 1^er^ janvier 2019 au 29 février 2020 soit 14 mois; et pour la seconde période pandémique du 1^er^ mars 2020 au 30 avril 2021 soit 14 mois.

## Résultats

Durant la pandémie (période 2) les activités menées se résument ainsi :

- environ 386 000 personnes ont été sensibilisées au coronavirus et à sa prévention à l'aide de focus groups, de causeries communautaires et d'entretiens téléphoniques;
- 143 visites à domicile ont été réalisées lorsque les cas présentaient une extrême vulnérabilité (Covid et maladie chronique, décès d'un proche, appui en vivres et autres, stigmatisation, refus du confinement);
- 240 situations concernant la prévention et le traitement des cas de stigmatisation et de discrimination ont été gérés;
- les ONG et associations ont été accompagnées sur les mesures barrières et les idées reçues sur la Covid-19; mais aussi les écoutes et la gestion des cas de VBG pour les personnes dépistées lors des consultations ou suite à un signalement.

Concernant les VBG, les équipes ont enregistré et suivi durant la même période 57 cas de viol, 7 agressions sexuelles et 171 agressions physiques, 125 situations de violence psychologique/émotionnelle, 35 cas de maltraitance et 7 mariages forcés. Ces données ont été comparées (Tableau I) à celles de la première période pré-pandémique, recueillies dans les mêmes conditions pour ce qui est des VBG.

**Tableau I:** Distribution des cas signalés de violences basées sur le genre (VBG) en 2019-2020 puis en 2020-2021, Grand Abidjan Distribution of reported cases of gender-based violence (GBV) in 2019-2020 and 2020-2021, Greater Abidjan

	Période 1 (janv. 2019_fév. 2020)					Période 2 (mars 2020_avril 2021)				
Type de VBG	Nb	Genre		Age		Nb	Genre Âge		Age	
		F	M	< 18 ans	18 et +		F	M	< 18 ans	18 et +
Viol	41	41	0	36	5	57	56	1	50	7
Agression sexuelle	4	4	0	3	1	7	7	0	6	1
Agression physique	139	135	4	0	139	171	161	10	0	171
Maltraitance	27	2	25	27	0	35	0	35	35	0
Mariage forcé	4	4	.	3	1	7	7	0	5	2
Violence psychologique	118	72	46	0	118	125	95	30	0	125
**TOTAL**	**333**	**258**	**75**	**69**	**264**	**402**	**326**	**76**	**96**	**306**

En valeur absolue, une augmentation du nombre de VBG a donc été relevée durant la période d’émergence de la pandémie de Covid-19. La proportion des viols, qui concernent quasi exclusivement le genre féminin, augmente légèrement : 14,2 % des VBG en 2020 vs 12,3 % en 2019. Il en est de même pour les agressions sexuelles et les mariages forcés. Les agressions physiques et les violences psychologiques sont aussi majoritairement subies par les femmes, mais plus fréquentes chez les plus de 18 ans. Seule la maltraitance est plus fréquente chez les garçons jeunes.

Les cas rapportés ont une distribution spatiale hétérogène sans lien apparent avec la fréquence des cas de Covid-19, comme le montre le Tableau II.

**Tableau II:** Distribution par commune (ou groupe de communes) des cas de VBG et de Covid-19 de mars 2020 à avril 2021, Grand Abidjan Distribution of GBV and Covid-19 cases by municipality (or group of municipalities) from March 2020 to April 2021, Greater Abidjan

Commune ou groupe de communes	Population estimée en 2021 [4]	Nb de cas de Covid-19 (p. 1 000 hab.)	Nb de cas de VBG (p. 1 000 hab.)
Adjamé et Attécoubé	739430	3654 (5)	48 (0,06)
Anyama	125275	368(2)	7 (0,05)
Cocody / Bingerville	573332	19 217(33)	23 (0,04)
Koumassi	505256	1 856 (4)	10 (0,02)
Port-Bouët	488 415	1 438 (3)	10 (0,02)
Treichville / Marcory	411118	10 448 (25)	47 (0,11)
Abobo	1 202261	2335 (2)	229 (0,19)
Yopougon	1 249 953	5 454 (4)	33 (0,02)
**Total des 11 communes**	**5295040**	**44 770(8)**	**407 (0,08)**

L’évolution du nombre de cas de VBG est différente selon les communes (Tableau III). Sous l'hypothèse d'une population stable (N.B. : il n'y a pas eu de recensement général de population entre 2014 et fin 2021 en Côte d'Ivoire), une augmentation significative des cas de VBG est constatée dans l'ensemble des communes étudiées du Grand Abidjan mais aussi au niveau du groupe de communes de « Treichville et Marcory » et dans celle d'Abobo pendant le début de la pandémie de Covid-19 et inversement une baisse significative à Anyama, Port-Bouët et Yopougon à la même période.

**Tableau III:** Évolution des cas de VBG selon la commune (ou groupe de communes) entre les deux périodes d’étude, Grand Abidjan Changes in GBV cases by municipality (or group of municipalities) between the two study periods, Greater Abidjan

Commune ou groupe de communes	Population estimée en 2021	Nb de cas de VBG de janvier 2019 à février 2020	Nb de cas de VBG de mars 2020 à avril 2021	Sens et proportion de variation	Degré de signification p)
Adjamé et Attécoubé	739430	46	48	+4,3	0,92
Anyama	125275	23	7	-69,5	0,006
Cocody et Bingerville	573332	29	23	-20,7	0,41
Koumassi	505256	9	10	+ 11,1	0,81
Port-Bouët	488 415	29	12	-58,6	0,01
Treichville et Marcory	411118	18	47	+ 161,1	5,10^-4^
Abobo	1 202261	101	229	+ 126,7	3,10^-12^
Yopougon	1 249 953	78	33	-57,7	3,10^-5^
**Total des 11 communes**	**5295040**	**333**	**407**	**+22,2**	**0,007**

## Discussion

Notre étude confirme l'importance des VBG dans le District du Grand Abidjan, de plus elle met en évidence une variation importante et contrastée de l'incidence de celles-ci à l'occasion du début de la pandémie de Covid-19 entre mars 2020 et avril 2021.

Néanmoins cette étude souffre de lacunes liées à l'absence de données de population annualisées fiables, à la variabilité de passation des questionnaires par les travailleurs sociaux issus de formations professionnelles variées, à la fréquentation et à l'attractivité variables des centres de santé selon les communes, à la non-insertion de 2 des 13 communes du Grand Abidjan (certes peu peuplées), à la probable amélioration des pratiques des travailleurs sociaux dans un processus encore récent pour eux au moment de l’émergence de la pandémie de Covid-19, à la difficulté des victimes à aborder ces sujets des VBG, ou encore à l'insuffisance des moyens de communication pour le suivi des cas contacts et des difficultés de coordination entre les structures de santé et sociales.

Il semble néanmoins bien avoir eu à Abidjan une aggravation globale des violences et des VBG pendant la pandémie de Covid-19, ce qui a été observé ou redouté ailleurs [1, 2, 3, 4, 5].

## Conclusion

La pandémie de Covid-19 a été marquée par une augmentation globale des cas de VBG dans le Grand Abidjan. Il est donc important de poursuivre cette action des travailleurs sociaux auprès des victimes, en partenariat avec les soignants, en améliorant la coordination (décloisonnement) entre secteurs social et sanitaire.

## Liens D'intérêts

Les auteurs ne déclarent aucun lien d'intérêt.

## Contribution Des Auteurs

BENIE BI Vroh Joseph : coordonnateur du Projet au niveau du ministère de la Santé

AMETHIER Solange : rédaction du manuscrit

SANGARE Baba : recueil des données du terrain

KROMAN Savané Sita : relecture et correction du manuscrit

KADJO KOUADIO Florence : relecture et correction du manuscrit

Daouda COULIBALY et CHERIF Djibril : coordination de la mise en œuvre de l'enquête décembre 2020. www.academie-medecine.fr/wp-content/uploads/2020/12/20.12.18-Covid-19-et-violences.pdf.
